# Advances on image interpolation based on ant colony algorithm

**DOI:** 10.1186/s40064-016-2040-9

**Published:** 2016-04-01

**Authors:** Olivier Rukundo, Hanqiang Cao

**Affiliations:** Department of Communications and Information Sciences, Tilburg University, Warandelaan 2, 5037 AB Tilburg, The Netherlands; Department of Electronic and Information Engineering, Huazhong University of Science and Technology, 1037 Luoyu Road, Wuchang, Wuhan, 430074 People’s Republic of China

**Keywords:** Global weight, Local weight, Ant colony optimization, High resolution, Image interpolation

## Abstract

This paper presents an advance on image interpolation based on ant colony algorithm (AACA) for high resolution image scaling. The difference between the proposed algorithm and the previously proposed optimization of bilinear interpolation based on ant colony algorithm (OBACA) is that AACA uses global weighting, whereas OBACA uses local weighting scheme. The strength of the proposed global weighting of AACA algorithm depends on employing solely the pheromone matrix information present on any group of four adjacent pixels to decide which case deserves a maximum global weight value or not. Experimental results are further provided to show the higher performance of the proposed AACA algorithm with reference to the algorithms mentioned in this paper.

## Background

Ant colony (optimization) algorithm, as most of the bio-inspired computational techniques, provides a very good solution to difficult optimization problems (Dorigo and Stutzle 2004). Techniques based on this nature-inspired approach have been widely applied in many engineering areas including image processing though their applications for image interpolation purposes are still few. The most common issue in image interpolation is the low-pass filtering process which reduces, to some degree, the resolution of interpolated image. However, high resolution image interpolation process has been a problem of prime importance in many fields due to its wide application in satellite imagery, biomedical imaging, particularly in military and consumer electronics domains.

In our previous work (Rukundo et al. 2011), ant colony optimization has been used to reinforce locally the traditional bilinear weighting scheme, in order to achieve a higher resolution interpolation. Furthermore, in Tian et al. (2011), it has been applied to classify each wavelet coefficient into one of the Gaussian component, positive exponential and negative exponential components, before estimating the parameters of each component, but this is beyond the scope of this paper.

The local weighting scheme used in our previous work improved the peak signal to noise ratio (PSNR). However, that scheme could not avoid the influence of isotropic weighting of the conventional bilinear. Such isotropic weighting is responsible for many blurring artefacts which make the interpolated image look smoother than the original, thus reducing its resolution. As a solution, we propose a global weighting scheme which employs the pheromone matrix information solely, present on any group of four adjacent pixels, to decide which case deserves a maximum global weight value or not.

 This paper is organized as follows: first section is the “Background”; second section introduces the “Ant colony (optimization) algorithm”; third section presents “The proposed global weighting scheme”; fourth section gives the “Experimental results” and last section the “Conclusion and recommendations”.

## Ant colony (optimization) algorithm

Dorigo and Gambardella (1997) introduced modifications in ant system (AS) to increase its performance (Dorigo et al. 1991), and be able to find a very good solution to difficult optimization problems. Such modifications are: (a) a different transition rule; (b) different pheromone trail updating rules: global and local; and (c) a candidate list to restrict the choice of the next node to visit (Dorigo and Gambardella 1997), (Dorigo and Stutzle 2004).

### Ant colony optimization state transition rule

**Fig. 1:**
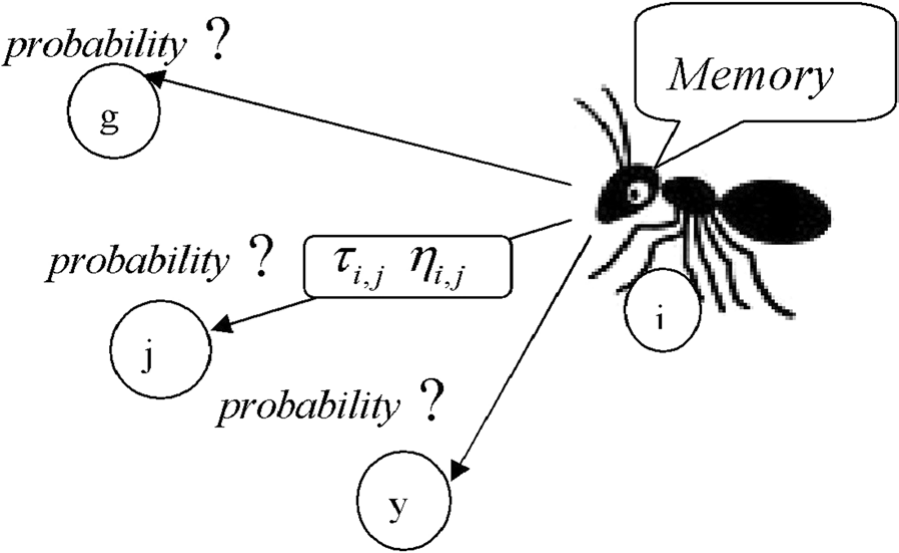
The selection of the node

Figure 1 shows an ant arriving in node *i*, while Table 1 shows the pseudocode of the implementation process. As shown in Fig. 1, the ant chooses the next node to move to as a function of the pheromone values $\tau_{i,j}$ and the heuristic values $\eta_{i,j}$ on the arcs connecting node *i* to the nodes *j* the ant has not visited yet.

**Table 1:** A pseudocode of implementation

Do initialization procedures
**for** each iteration n = 1:N **do**
**for** each construction_step l = 1:L **do**
**for** each ant k = 1:K **do**
Select and go to next pixel according to **exploitation** and **exploration** mechanism
Apply the local pixel’s updating rule
**end**
**end**
Apply the global updating rule using the best visited pixels’ pheromones
**end**
Decide a global weight based the best visited pixel’s pheromone information
**End**

 The next node is chosen among the non-visited nodes according to the following pseudorandom proportional rule, in which the transition probability depends on a random variable *q* that is uniformly distributed over [0, 1] and a control parameter $q_{0} (0\leqq_{0}\leq1)$

$$j = \left\{ {\begin{array}{*{20}ll} {\arg \max_{{f \in \Omega_{i}^{k} }} \{ \tau_{if} (\eta_{if} )^{\beta } \}}, \hfill & \quad {if}& {q \le q_{0} \,(Exploitation)} \hfill \\ J, \hfill & \quad {else}&\;{(Exploration)} \hfill \\ \end{array} } \right.$$

If $q \le q_{0} ,$ then the transition that maximizes $\tau_{i,j} (\eta )^{\beta }$ is chosen otherwise, and the probabilistic decision rule, Eq. (1) with $\alpha = 1,$ is used. The value of $q_{0}$ determines the degree of exploration of the ants: with probability $q_{0} ,$ the ant chooses the transition with the highest $\tau_{i,j} (\eta )^{\beta } ,$ while with probability $1 - q_{0} ,$ it performs a biased exploration of the edges. The balance between biased exploration and pheromone exploitation can be tweaked by adjusting the value of $q_{0} .$

1 $$p_{i,j}^{k} = \frac{{(\tau_{i,j}^{\alpha } )(\eta_{i,j}^{\beta } )}}{{\sum\nolimits_{{f \in \Omega_{i}^{k} }} {(\tau_{i,f}^{\alpha } )(\eta_{i,f}^{\beta } )} }}\quad if\;\begin{array}{*{20}c} {j \in \Omega_{i}^{k} } \\ {\alpha = 1} \\ \end{array}$$

In Eq. (1), $\tau_{i,j}$ is the amount of pheromone deposited for transition from state *i* to state *j*. $\alpha \ge 0$ and $\beta \le 1$ are parameters to control the influence of $\tau_{i,j}$ and $\eta_{i,j} ,$ respectively. $\eta_{i,j}$ is the desirability of the state transition $i,j$ and is equal to $1 /d_{i,j}$ where *d* is the distance in Traveling Salesman Problem (TSP). The $\Omega_{i}^{k}$ represents an acceptable neighborhood for an ant *k* while being at city *i* (here, the probability of selecting a city outside acceptable neighborhood is zero).

### Ant colony optimization pheromone trail updating

#### Local updating

Ants use a local pheromone update rule after leaving the arc $(i,j)$ during the construction process as follows:

2 $$\tau_{i,j} \leftarrow (1 - \varphi )\tau_{i,j} + \varphi\Delta \tau_{0}$$

where $\varphi ,\,(0 < \varphi < 1),\,\tau_{i,j}$ is the current value of the pheromone trails at $i,j,$ and $\tau_{0}$ is the initial value for the pheromone trails.

#### Global updating

The best ant reinforces its tour by depositing additional pheromone trails along the tour length

3 $$\tau_{i,j} \leftarrow (1 - \rho )\tau_{i,j} + \rho \Delta \tau_{i,j}^{bs} ,\quad \forall (i,j) \in T^{bs}$$

where $\Delta \tau_{i,j}^{bs} = 1 /C^{bs}$ (here, $C^{bs}$ is the best-so-far tour) and $\rho$ represent the pheromone evaporation. For this case, the computational complexity is reduced from $\partial (n^{2} )$ to $\partial (n)$ (where *n* is the instance being solved) by only performing the pheromone updates to the arc of $T^{bs}$ (not to all arcs as in ant system).

### Ant colony optimization candidate list

A candidate list is a list of preferred cities to visit. Instead of examining all the cities, non-visited cities are examined first. This list is ordered by increasing distance and helps to select the next city when other cities have been visited.

### Previous works on ant colony optimization for image interpolation

Tian et al. (2011) proposed a wavelet-based technique, employing ant colony optimization, for image interpolation. Firstly, a J-level wavelet decomposition was applied on the input low-resolution image. The proposed TCEM model was used to formulate the statistical distribution of the above J-level wavelet coefficients and the model parameters of the proposed TCEM model were estimated using the ant colony optimization. The distribution of the desired high-pass filtered wavelet coefficient in the 0th level subband was estimated and then generated the 0th level wavelet coefficients. Finally, the (J + 1)-level inverse wavelet transform was applied to produce the final reconstructed image.

Experiments, using grayscale test images, were conducted to compare the performance of the proposed approach with that of algorithm mentioned. However, the basic wavelet based technique idea used in Tian et al. (2011) is beyond the scope of this paper.

Rukundo et al. (2011) applied ant colony optimization pheromone information to supplement the bilinear isotropic weighting scheme in order to achieve improved (or higher) resolution results. Here, ant colony optimization was used to construct the pheromone matrix and then find the pixels on which more pheromone information was deposited. This pheromone matrix information was used to strengthen the traditional bilinear algorithm weighting scheme shown in Eq. (4).

4 $$P_{x,y} = w_{4} P_{u,v} + w_{2} P_{u,v + 1} + w_{3} P_{u + 1,v} + w_{1} P_{u + 1,v + 1}$$

where $(x,y)$ represents the coordinates of unknown-value location, $(u,v),\,(u + 1,v),\,(u,v + 1),$ and $(u + 1,v + 1)$ are coordinates of each of the four (known-value) pixels surrounding $(x,y)$ location.

5 $$\begin{aligned} w_{1} & = \{ [x - u][y - v]\} ,w_{2} = \{ [x - (u + 1)][y - (v)]\} , \\ w_{3} & = \{ [x - (u)][y - (v + 1)]\} ,w_{4} = \{ [x - (u + 1)][y - (v + 1)]\} \\ \end{aligned}$$

Our previous OBACA algorithm used the pheromone matrix information (obtained after both updates were performed), to create additional but necessary weights. In other words, the pheromone matrix information was assigned on every pixel with reference to the neighboring pixels intensity variations. The pheromone information attached to each pixel became its weight, to locally supplement the traditional bilinear weighting scheme, as shown in Eq. (6),

6 $$P_{x,y} = p_{4} w_{4} P_{u,v} + p_{2} w_{2} P_{u,v + 1} + p_{3} w_{3} P_{u + 1,v} + p_{1} w_{1} P_{u + 1,v + 1}$$

where $p_{4} ,p_{2} ,\,p_{3} ,\,p_{1}$ represent the pheromone information at $(u,v),(u + 1,v),\,(u,v + 1)$ and $(u + 1,v + 1),$ respectively. In this way, OBACA achieved higher PSNR or image quality when compared to that of the traditional bilinear. However, it could not avoid the influence of isotropic weighting of the conventional bilinear which is responsible for smoothing/blurring the output/interpolated images, thus reducing their resolutions. This is the aim of our research to develop a global weighting scheme whose basic function is shown by Eq. (7), where $w_{g}$ the global weight produced by ant colony optimization algorithm.

7 $$P_{x,y} = w_{g} \left( {w_{4} P_{u,v} + w_{2} P_{u,v + 1} + w_{3} P_{u + 1,v} + w_{1} P_{u + 1,v + 1} } \right)$$

Furthermore, the developed weighting scheme considers “the characteristics of pheromone information locality” in order to assign that global weight thus achieves a higher resolution image interpolation.

## The proposed global weighting scheme

**Fig. 2:**
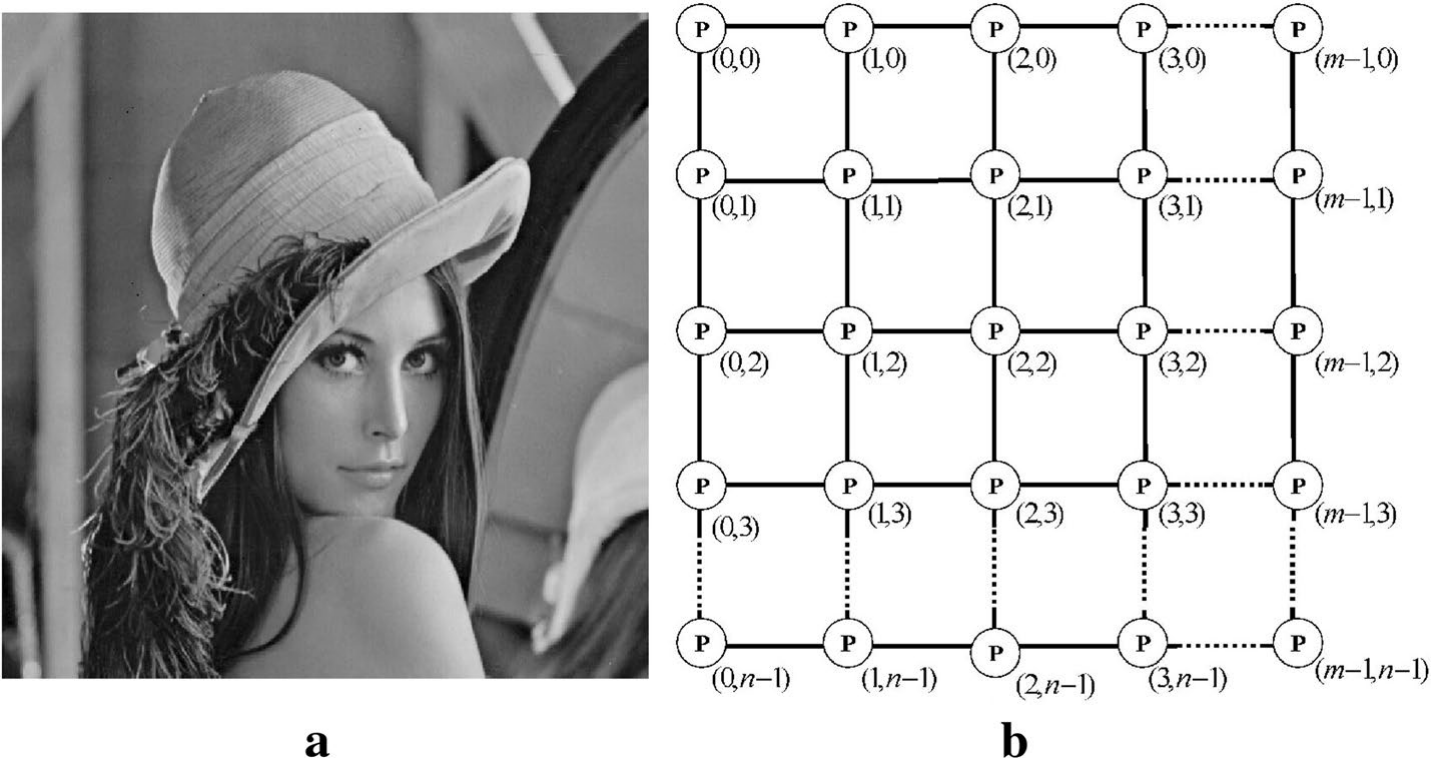
**a** Lenna image. **b** Example of a two-dimensional representation with the pheromone information P assigned on each image pixel location

As shown by Eq. (7), $w_{g}$ is the global weight which is applied to a group of pixels surrounding directly an unknown-value location. This global weight is a function of pheromone matrix information on that/each group of four pixels (see Fig. 2b). Now, the problem is how to find the pheromone information present on each group. To solve this problem, we started by constructing the pheromone matrix and then we checked possibilities of the partial or full simultaneous presence of the pheromone trails information at the four pixels/locations surrounding directly the unknown-value location $P_{x,y}$ .

### Pheromone matrix construction

**Fig. 3:**
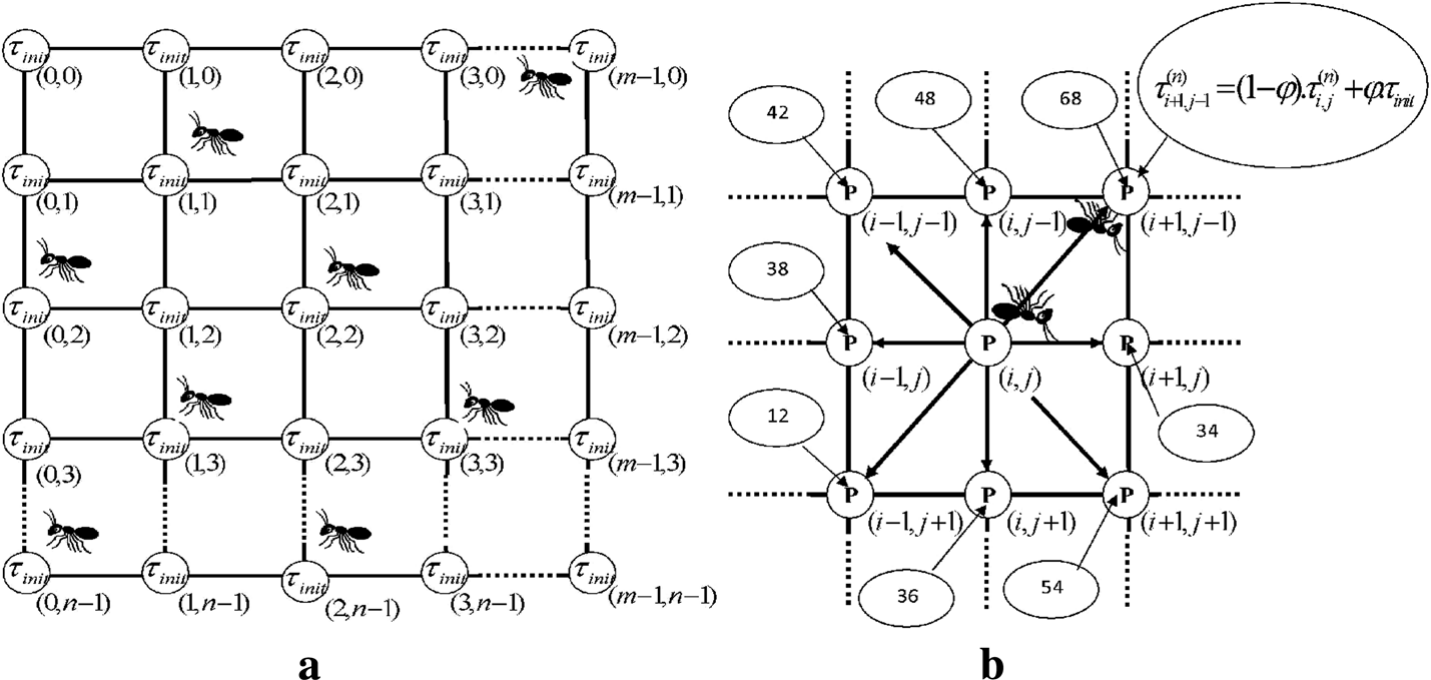
**a** Initial pheromone matrix look. Initially, the pheromone value for each matrix element is set to a non-zero constant, **b** the 8-connectivity neighborhood and possible ant’s direction movement

Initially a pheromone value $\tau_{init}$ is assigned to every pixel location (see Fig. 3a) and, ants are randomly distributed on image before moving artificial ants for a certain number of steps on that image.

The ants’ movements are/will be steered by the intensity variation of the grayscale pixels (within a permissible range of 8-connectivity neighborhood around $P_{(i,j)} ,$ as shown in Fig. 3b.

The *n*th ant’s move from a departing pixel’s position $\left( {i_{0} ,j_{0} } \right)$ located within acceptable (8-connectivity) neighborhood depends on the attractiveness $\eta_{i,j}$ of any adjacent pixel [e.g.$i,j$] and the pheromone trail information $\tau_{i,j}^{(n - 1)}$ on it. Therefore, an ant will make a *n*th move from any pixel $\left( {i_{0} ,j_{0} } \right)$ to an adjacent pixel $(i,j)$ according to the pseudorandom proportional rule. The transition probability will be given by Eq. (8)

8 $$p_{{(i_{0} ,j_{0} ),(i,j)}}^{(n)} = \frac{{\left( {\tau_{i,j}^{(n - 1)} } \right)^{\alpha } \left( {\eta_{i,j}^{(n - 1)} } \right)^{\beta } }}{{\sum\nolimits_{{(i,j) \in \Omega (i_{0} ,j_{0} )}} {\left( {\tau_{i,j}^{(n - 1)} } \right)^{\alpha } \left( {\eta_{i,j}^{(n - 1)} } \right)^{\beta } } }}$$

where $\tau_{i,j}^{(n - 1)}$ is the pheromone value on the pixel $(i,j);\,\Omega_{{(i_{0} ,j_{0} )}}$ is an acceptable neighborhood for the pixel $(i_{0} ,j_{0} );\,\eta_{i,j}$ is the heuristic information on the pixel $(i,j);$ the pheromone and heuristic information constants $\alpha$ and $\beta$ are always positive. Each time an ant visits a pixel, it automatically performs a local update on the adjacent pixel. The amount of pheromone on the pixel $(i,j)$ at the *n*th iteration, $\tau_{i,j}^{(n)}$ is calculated using the local pheromone update Eq. (9)

9 $$\tau_{i,j}^{(n)} = (1 - \varphi ) \cdot \tau_{i,j}^{(n)} + \varphi \cdot \tau_{init}$$

where $\varphi \in [0,1]$ is the pheromone decay coefficient. In each iteration, the pheromone trail value changes because local updates are provided together with the solution construction process. As mentioned, the ant’s permissible range of movements is situated within the 8-connectivity neighborhood, see Fig. 3b. An ant can move to an adjacent pixel on the condition that it moves to a pixel that was not recently visited by any other ants. This condition is backed by an artificial memory assigned to each ant so that it can keep records of every visited pixel. After all ants have completed the construction process, a global pheromone update is performed only on the pixels that have been visited by at least one ant according to Eq. (10)

10 $$\tau_{i,j}^{(n)} = (1 - \rho ) \cdot \tau_{i,j}^{(n - 1)} + \rho \cdot \sum\limits_{K = 1}^{K} {\Delta \tau_{i,j}^{(k)} }$$

where $\Delta \tau_{i,j}^{(k)}$ is the amount of pheromone deposited by the *k*th ant on the pixel $(i,j).$ This amount $\Delta \tau_{i,j}^{(k)}$ is equal to the average of heuristic information associated with the pixels that belong to the tour of the *k*th ant if the pixel $(i,j)$ was visited by the *k*th ant in its current tour, otherwise the amount $\Delta \tau_{i,j}^{(k)} = 0;\,\rho$ is the pheromone evaporation rate; *K* is the number of ants which can be determined by Eq. (11).

11 $$K = \sqrt {input\,image\,width\,*\,input\,image\,height}$$

As the value for $\Delta \tau_{i,j}^{(k)}$ depends on the heuristic information, $\eta_{i,j}$ at the $(i,j)$ can be given by Eq. (12)

12 $$\eta_{(i,j)} = \frac{{V_{c} (P_{i,j} )}}{{V_{\rm max } }}$$

where $P_{(i,j)}$ is the intensity value of a pixel at $(i,j),\,V_{\rm max }$ is the normalization factor; $V_{c} (P_{i,j} )$ is the function that operates on the 8-connectivity pixels neighborhood. The value of that function depends on the variation of the pixel’s intensity values and can be given by Eq. (13)

13 $$\begin{aligned} V_{c} (P_{i,j} ) & = \left| {P_{(i - 1,j - 1)} - P_{(i + 1,j + 1)} } \right| + \left| {P_{(i + 1,j - 1)} - P_{(i - 1,j + 1)} } \right| \\ & \quad + \,\left| {P_{(i,j - 1)} - P_{(i,j + 1)} } \right| + \left| {P_{(i - 1,j)} - P_{(i + 1,j)} } \right| \\ \end{aligned}$$

### Max and mean global weighting scheme

This is a very important step of our proposed algorithm. The max function returns the greatest element value in a data set whereas the mean returns the average of elements values. Equation (10) gives the pheromone value or level on mostly visited pixel locations. However, it has been experimentally upgraded to Eq. (14) in order to yield viewable results.

14 $$p_{(i,j)} = \exp \left( {\tau_{i,j}^{n} } \right)$$

**Fig. 4:**
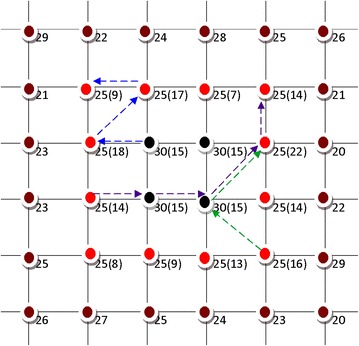
The estimation of an ant to select the next pixel

Intuitively, assigning a direct the global weight, as shown by Eq. (7), would not permit to reduce the low-pass filtering process which reduces image resolution. However, in order to achieve higher resolution or quality image interpolation, we have introduced a weighting scheme which employs solely the Eq. (14) pheromone information, present on any group of four adjacent pixels, to decide which interpolated pixel that needs the maximum global weight or not. Now, with reference to Fig. 4 and Eq. (12), we can estimate the desirability of an ant to select the next pixel using the function $V_{c} (P_{i,j} ).$

For instance, Fig. 4 shows that an ant belonging to the upper left black pixel dot 30 (15), where 30 is the pixel value and 15 is $V_{c} (P_{i,j} )$ value, has eight choices in which the best one (i.e. one having the highest $V_{c} (P_{i,j} )$ value) is acceptable, if and only if it has not been chosen by any (other) ants. In this way, the ant will follow the blue, purple or green arrows depending on where it was initially put or randomly distributed. Their moves along those arrows shows that the pheromone information can be present (at least) at three, two and one locations in a group (of four pixels), because every time an ant moves, it drops the pheromone trails on the visited pixel or location.

**Fig. 5:**
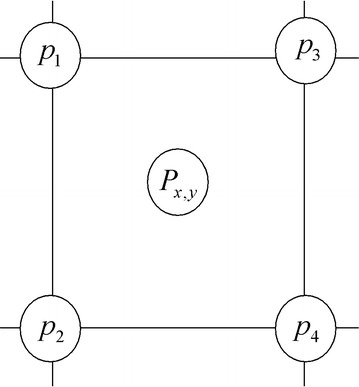
Pheromones trails around the unknown-value location $P_{x,y}$

Now, the next problem is how reasonably to assign the global weight in each (simultaneous pheromone trails presence) case. To settle this, we label *p*_1_, *p*_2_, *p*_3_ and *p*_4_ as the pheromone information on each pixel position surrounding directly the unknown-value location $P_{x,y}$ shown in Fig. 5, and that *A*, *B*, *C* and *D*are different levels or value that *p*_1_, *p*_2_, *p*_3_ and *p*_4_ can have. Let us now make *S* a set containing the pheromone trails information *p*_1_, *p*_2_, *p*_3_ and *p*_4_ as shown by Eq. (15).

15 $$S = \left[ {p1,p2,p3,p4} \right]$$

In order to reduce reasonably the low-pass filtering process in our algorithm we used max and mean functions to assign reasonably the global weight with reference to possible partial or full simultaneous presence of the pheromone trails information at the four locations surrounding directly the unknown-value location $P_{x,y}$.

#### **Assumption 1**

When $p_{1} = p_{2} = p_{3} = p_{4} = A$ (all pheromone trails information around $P_{x,y}$ have the same level).

16 $$Max(S_{1} ) = A$$

And

17 $$Mean(S_{1} ) = A$$

Here, there is no special requirement for assigning the highest level of pheromone trails. Therefore, any of two weighting procedures can be used, my experimental choice is given by Eq. (18)

18 $$w_{g} = Mean(S_{1} )$$

#### **Assumption 2**

When $p_{1} = p_{2} = p_{3} = A\,and\,p_{4} = B$ (one of the four pheromone trails information around $P_{x,y}$ has a different level).

19 $$\left\{ {\begin{array}{*{20}c} {Max(S_{2} ) = A} & \quad {if\;p3 > p4} \\ {Max(S_{2} ) = B} & \quad {otherwise} \\ \end{array} } \right.$$

And

20 $$Mean(S_{2} ) \ne A \ne B$$

Here, a highest level of pheromone trails can be assigned reasonably only when $p_{3} > p_{4} ,$ since it is only in this case where you can find one location having the lowest pheromone trail level. For this reason, the global weight is given by Eq. (21)

21 $$w_{g} = Max(S_{2} )$$

#### **Assumption 3**

When $p_{1} = p_{2} = A,p_{3} = C\,and\;p_{4} = B$(only two of the four pheromone trails information around $P_{x,y}$ have the same level).

22 $$\left\{ {\begin{array}{*{20}l} {Max(S_{3} ) = A} \hfill & \quad {if} \hfill & {p2 > \{ p3,p4\} } \hfill \\ {Max(S_{3} ) = C} \hfill & \quad {if} \hfill & {p2 < p3} \hfill \\ {Max(S_{3} ) = B} \hfill & \quad {if} \hfill & {p2 < p4} \hfill \\ \end{array} } \right.$$

And

23 $$Mean(S_{3} ) \ne A \ne B \ne C$$

A highest level of pheromone trails can be assigned reasonably only when $p_{2} > p_{3} \,\& \,p_{2} > p_{4} ,$ because it is in this case where you can only find a biggest number of locations with the highest pheromone trail level, at the same time. Therefore, the max weighting is given by Eq. (24)

24 $$w_{g} = Max(S_{3} )$$

#### **Assumption 4**

When $p_{1} = A,p_{2} = D,p_{3} = C\,and\,p_{4} = B$ (all pheromone trails information around $P_{x,y}$ have different levels).

25 $$\left\{ {\begin{array}{*{20}l} {Max(S_{4} ) = A} \hfill & \quad {if} \hfill & {p1 > \{ p2,p3,p4\} } \hfill \\ {Max(S_{4} ) = D} \hfill & \quad {if} \hfill & {p2 > \{ p1,p3,p4\} } \hfill \\ {Max(S_{4} ) = C} \hfill & \quad {if} \hfill & {p3 > \{ p2,p1,p4\} } \hfill \\ {Max(S_{4} ) = B} \hfill & \quad {if} \hfill & {p4 > \{ p2,p3,p1\} } \hfill \\ \end{array} } \right.$$

And

26 $$Mean(S_{4} ) \ne A \ne B \ne C \ne D$$

Here, a mean-based weighting procedure is preferable in order to avoid any possible influence of a strong noise intrusion. Therefore, the global weight is given by Eq. (27)

27 $$w_{g} = Mean(S_{4} )$$

#### **Assumption 5**

When $p_{1} = p_{2} = A,p_{3} = p_{4} = B$ (every two pheromone trails information around $P_{x,y}$ have the same level).

28 $$\left\{ {\begin{array}{*{20}l} {Max(S_{5} ) = A} \hfill & \quad {if} \hfill & {p2 > p3} \hfill \\ {Max(S_{5} ) = B} \hfill & \quad {if} \hfill & {p2 < p3} \hfill \\ \end{array} } \right.$$

And

29 $$Mean(S_{3} ) \ne A \ne B$$

A highest level of pheromone trails can be assigned reasonably only when $p_{2} > p_{3} \& p_{2} < p_{3}$ and therefore, the max weighting is given by Eq. (29)

30 $$w_{g} = Max(S_{5} )$$

### Parameter selection

The ant colony optimization has many parameters that need to be properly selected in order to increase the strength of the proposed algorithm. In this regard, the proper selection of parameters $\alpha$ and $\beta$ helps to determine the relative influence of the pheromone trail and the path visibility. Information in image content is always more important than in pheromone trail, thus, $\beta > \alpha$ is a general selection and our experiments adopt $\alpha = 1,\,\beta = 2$ as in Tian et al. (2011). Other parameters such as $\tau_{init} ,\,\varphi$ and $\rho$ determine the pheromone trail change, where $\tau_{init}$ is the initial value for the pheromone trails and this value is always non-zero though very close to zero. $\varphi$ and $\rho$ are adaptation parameters. Our experiment adopt $\tau_{init} = 0.0001,\,\varphi = 0.00001$ and $\rho = 0.1.$ Finally, the number of iterations, ants and memory size are also important parameters which deserve a proper selection. Note that a large number of iterations tend to increase the image edges sharpness but at very high computational load plus a possibility of generating too many false edge pixels. Furthermore, with a higher number of iterations, the change in image quality is not significant as it can be imagined intuitively. Artificial ant’s memory size can be determined by Eq. (31) to ensure that each ant is assigned a big enough memory to record all ‘*visitable*’ pixels.

31 $$Memory\;size = \frac{m*n}{K}$$

Experimentally, we found that the number of iterations ranging from 1 to 10, the number of ants given by Eq. (11) and the memory size given by Eq. (31) are adequate parameter values for yielding interpolated images in their most pleasant way within a tolerable computational load.

### Summary of the proposed algorithm

**Fig. 6:**
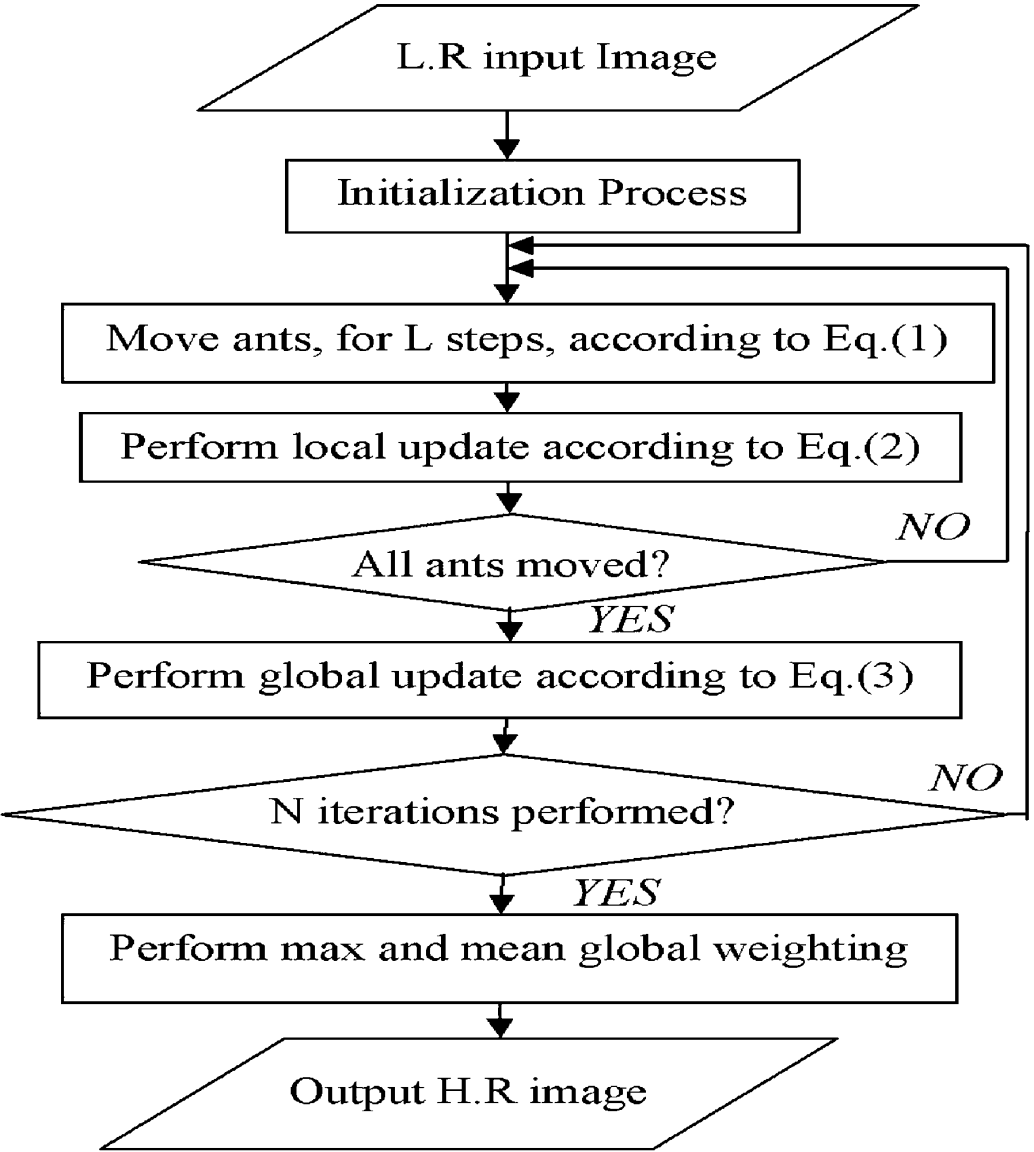
Summary of the proposed algorithm

Figure 6 shows a summary of the proposed AACA algorithm. The proposed algorithm includes two important steps namely, the pheromone matrix construction and global weighting scheme.

The proposed approach provides better results than our previously proposed methods as well as the interpolation algorithms mentioned in this paper. The novelty in this approach is a weighting scheme involved in its process. This scheme is based on global weight assignment rather than local. It uses the max and mean functions to adapt adequately the global weight to an interpolated pixel with reference to partial or full simultaneous presence of the pheromone trails information (at the four locations). This is done to reduce reasonably the low-pass filtering process thus achieve a HR image interpolation.

## Experimental results

**Fig. 7:**
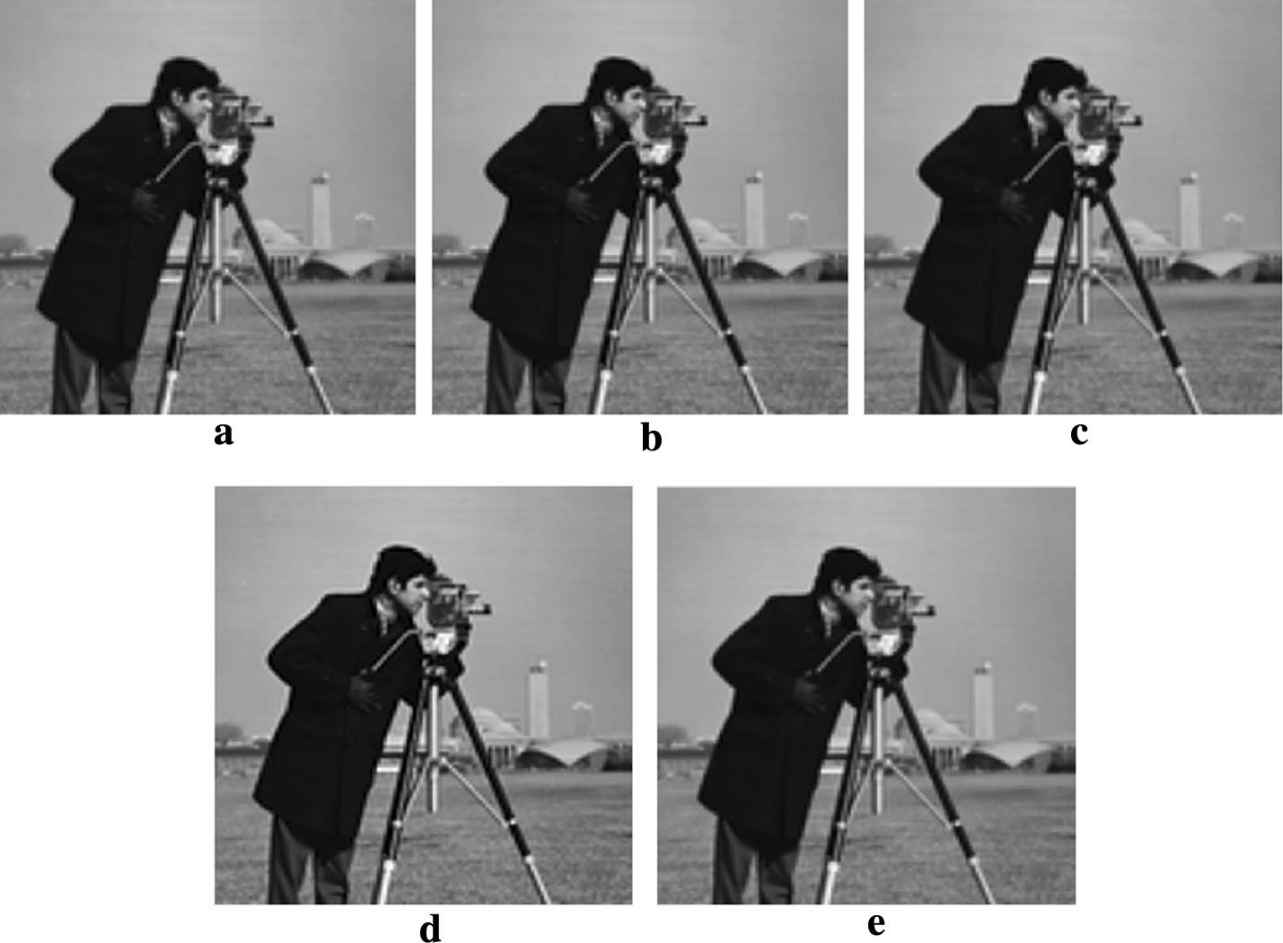
**a** Interpolated image by AACA, **b** interpolated image by NNV, **c** interpolated image by OBACA, **d** interpolated image by bicubic, and **e** interpolated image by bilinear algorithm

**Fig. 8:**
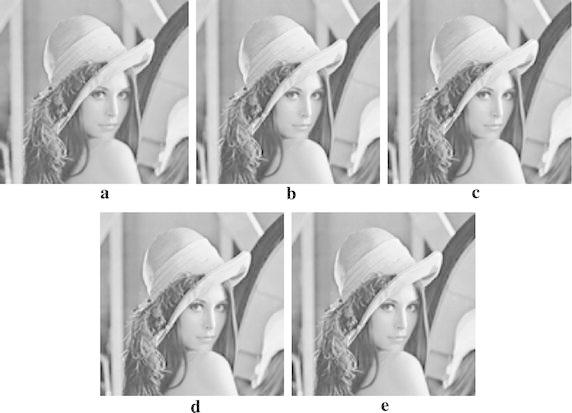
**a** Interpolated image by AACA, **b** interpolated image by NNV, **c** interpolated image by OBACA, **d** interpolated image by bicubic, and **e** interpolated image by bilinear algorithm

**Fig. 9:**
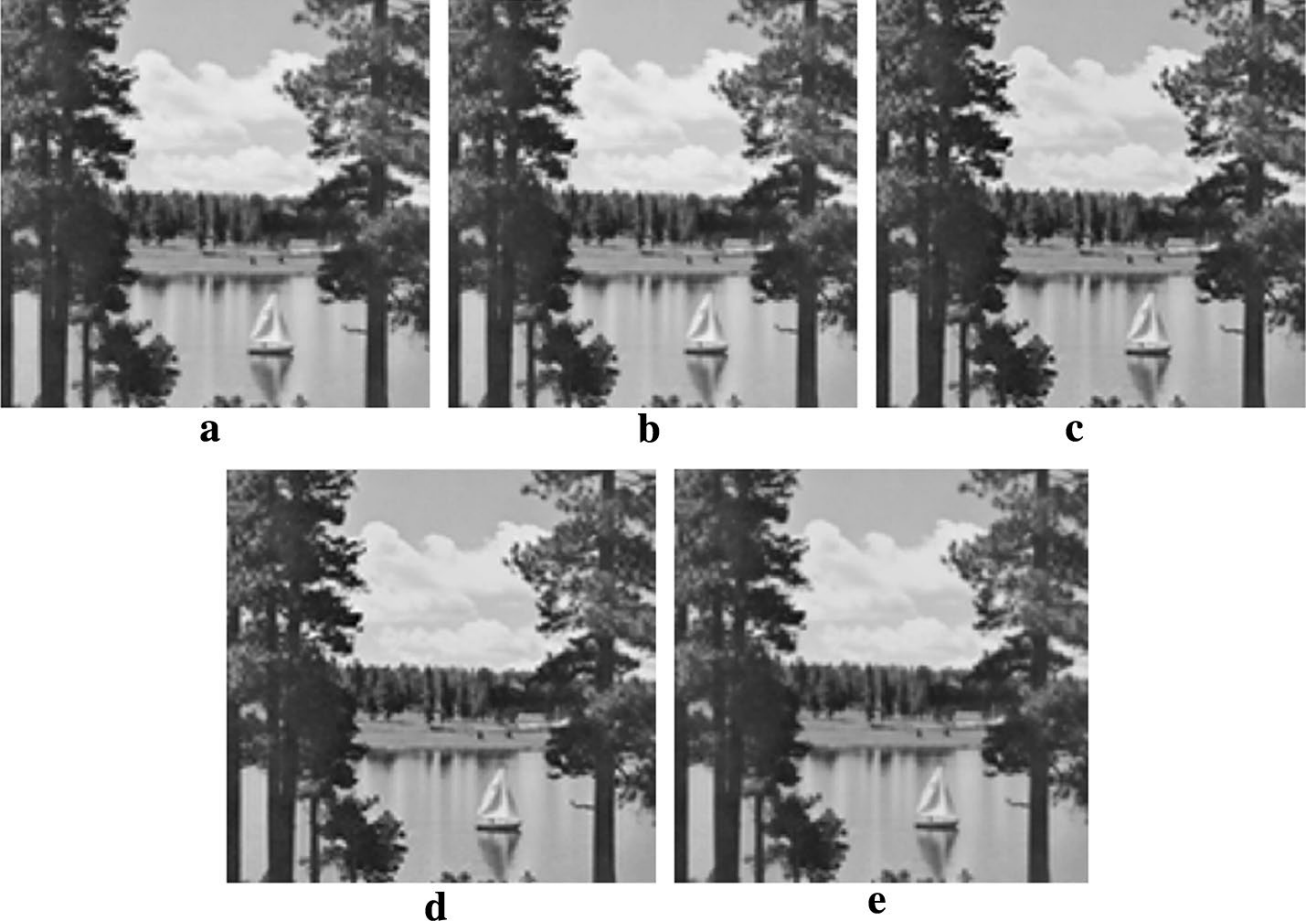
**a** Interpolated image by AACA, **b** interpolated image by NNV, **c** interpolated image by OBACA, **d** interpolated image by bicubic, and **e** interpolated image by bilinear algorithm

**Fig. 10:**
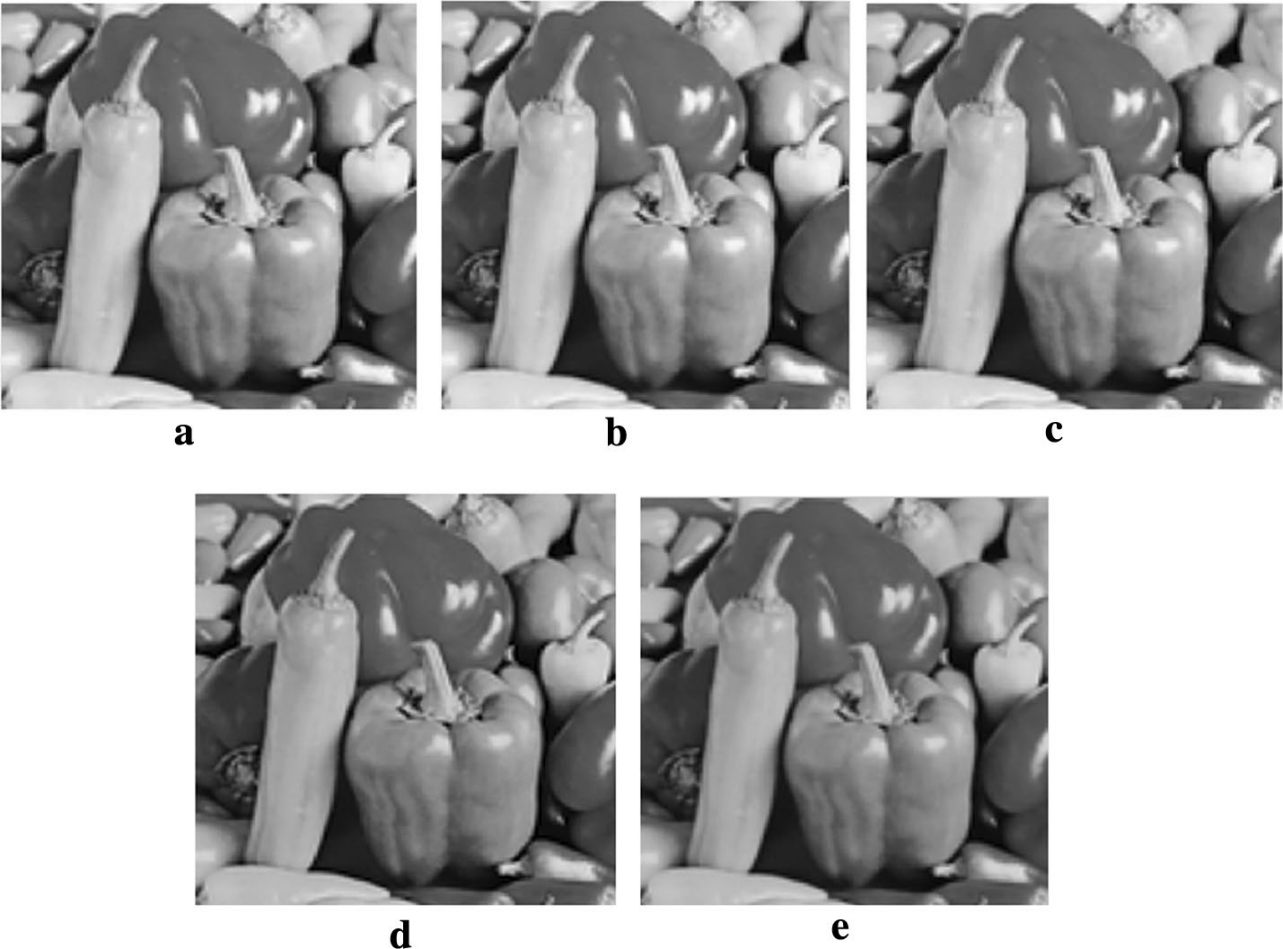
**a** Interpolated image by AACA, **b** interpolated image by NNV, **c** interpolated image by OBACA, **d** interpolated image by bicubic, and **e** interpolated image by bilinear algorithm

**Fig. 11:**
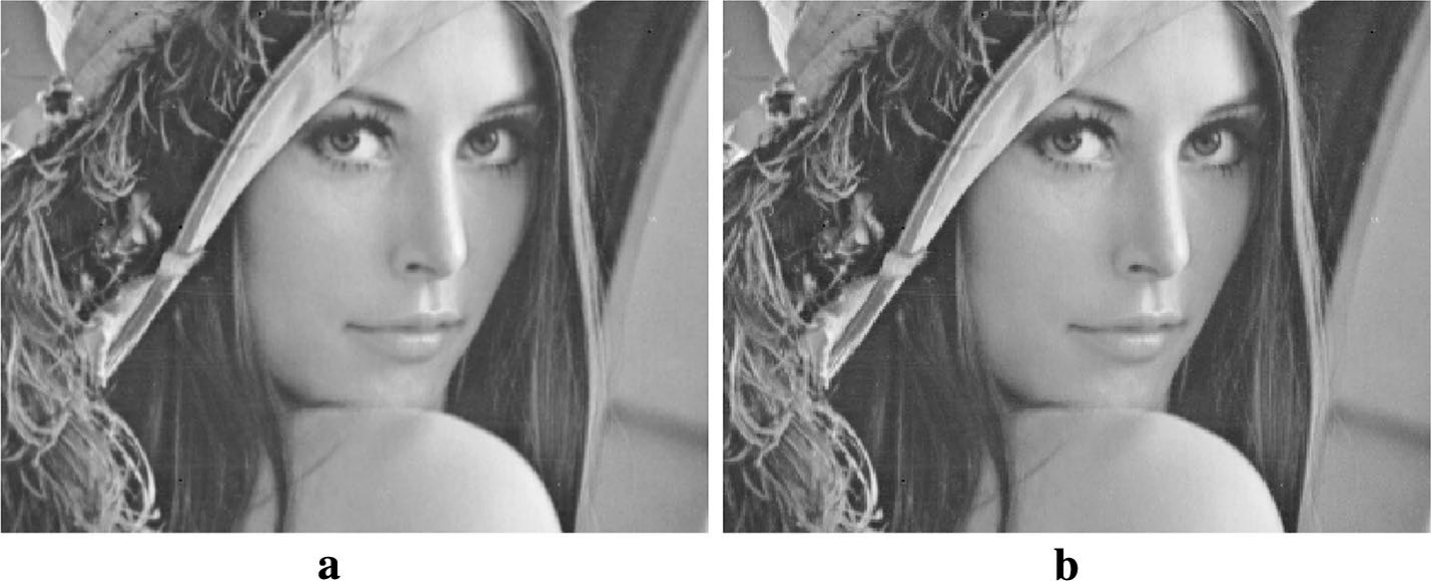
**a** Interpolated image by AACA (PSNR = 39.2180, MSE = 7.7853), **b** interpolated image by NEDI (PSNR = 37.1192, MSE = 12.6229)

**Fig. 12:**
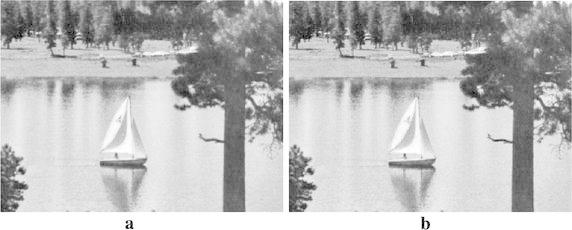
**a** Interpolated image by AACA (PSNR = 37.9615, MSE = 10.3976), **b** interpolated image by NEDI (PSNR = 35.9885, MSE = 16.3769)

**Fig. 13:**
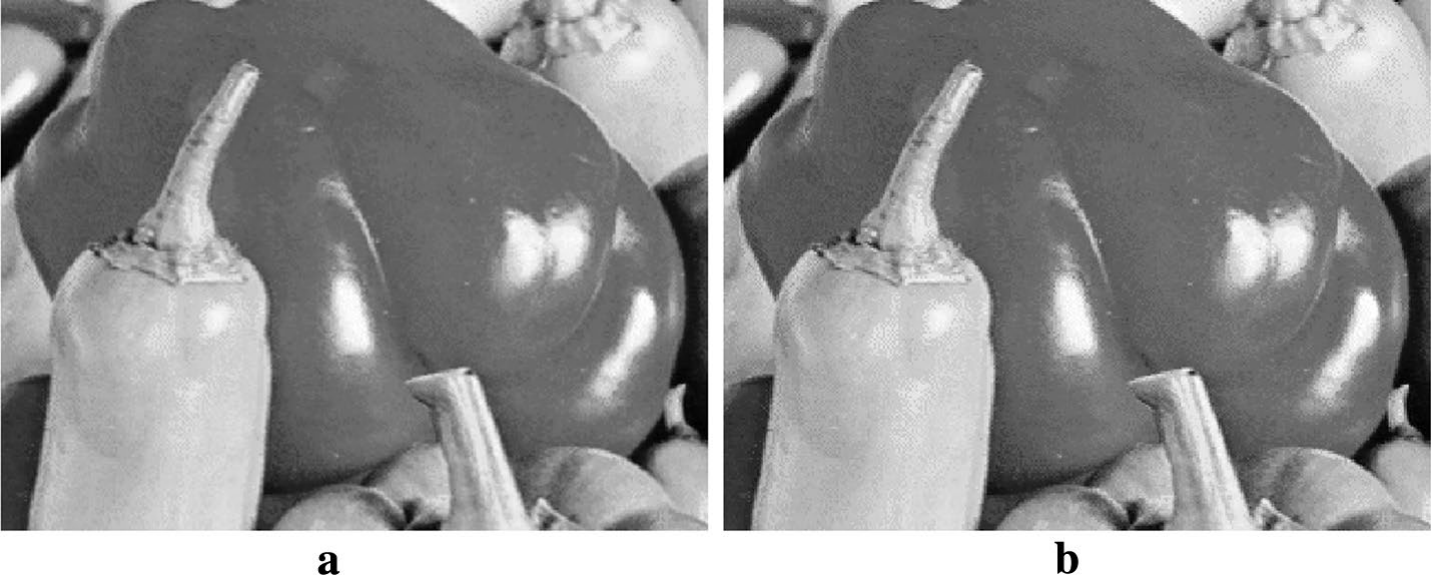
**a** Interpolated image by AACA (PSNR = 40.1928, MSE = 6.2201), **b** interpolated image by NEDI (PSNR = 37.8635, MSE = 10.6348)

**Fig. 14:**
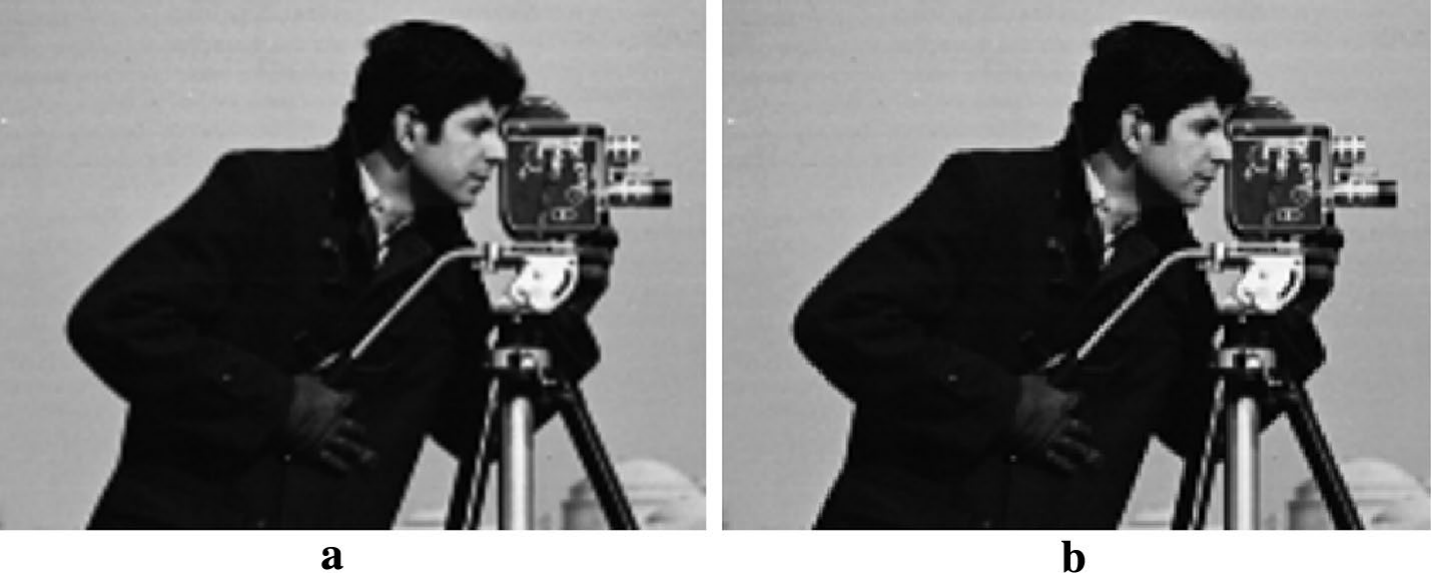
**a** Interpolated image by AACA (PSNR = 40.1144, MSE = 6.3334), **b** interpolated image by NEDI (PSNR = 37.4861, MSE = 11.6003)

This section presents the results from the experiments conducted. The performance of the proposed approach was compared with that of the previous works, two traditional interpolation algorithms executed by MATLAB toolbox and new edge directed interpolation algorithm proposed/whose details are given in Li and Orchard (2001). Two image quality assessment measures used are: Peak Signal to Noise Ratio (PSNR) and Mean Squared Error (MSE). A higher PSNR would normally indicate the higher quality of the output image whereas a higher MSE would indicate the interpolator’s weakness to reconstruct faithfully an image. Tables 2 and 3 provide the MSE and PSNR values for interpolated *Lenna*, *Cameraman*, *Lake* and *Peppers* test images. However, Figs. 7, 8, 9 and 10 show these images after being interpolated using the bilinear, bicubic, OBACA, Nearest Neighbor Value Interpolation (NNV) proposed in Rukundo and Cao (2012) and AACA interpolation algorithms presented in this paper. Furthermore, Figs. 11, 12, 13 and 14 show the portions of *Lenna*, *Lake*, *Peppers* and *Cameraman* images interpolated using New Edge Directed Interpolation (NEDI) and AACA. The PSNR and MSE were provided in the caption of Figs. 11, 12, 13 and 14.

**Table 2:** MSE of the interpolated images (128 × 512)

Images	Methods	Values
Cameraman	Bilinear	25.2190
	Bicubic	24.9344
	OBACA	21.9463
	NNV	20.9192
	AACA	17.4473
Lenna	Bilinear	23.0509
	Bicubic	22.1158
	OBACA	15.9800
	NNV	14.9963
	AACA	10.6305
Lake	Bilinear	43.2509
	Bicubic	42.5583
	OBACA	39.8727
	NNV	38.7641
	AACA	33.6966
Peppers	Bilinear	27.1935
	Bicubic	26.8901
	OBACA	22.9027
	NNV	21.2183
	AACA	17.7068

**Table 3:** PSNR of the interpolated images (128 × 512)

Images	Methods	Values (dB)
Cameraman	Bilinear	34.1135
	Bicubic	34.1628
	OBACA	34.7172
	NNV	35.5267
	AACA	35.7135
Lenna	Bilinear	34.5039
	Bicubic	34.6838
	OBACA	36.0950
	NNV	36.1891
	AACA	37.8653
Lake	Bilinear	31.7708
	Bicubic	31.8410
	OBACA	32.1240
	NNV	32.1377
	AACA	32.8549
Peppers	Bilinear	33.7862
	Bicubic	33.8349
	OBACA	34.5319
	NNV	34.5559
	AACA	35.6494

## Conclusions and recommendations

An advance on image interpolation based on ant colony algorithm has been presented in this paper. Unlike our previous OBACA algorithm, which employed a local weighting scheme, the presented AACA algorithm used however a novel global weighting scheme. The strength of the proposed global weighting depended on employing solely the pheromone matrix information, present on any group of four adjacent pixels, to decide which case deserves a maximum global weight value or not. The use of max and mean global weight values has shown that the proposed AACA algorithm is able to reduce the interpolation errors with reference to the original image. This was proved by the experiments conducted on full and partial *Cameraman*, *Lenna*, *Lake* and *Peppers* test images. More particularly, it has been shown by higher quality/resolution images yielded by AACA algorithm than other algorithms mentioned in this paper. The future development of the proposed approach may be devoted to settling the computational load issue by restricting the movements of ants on some parts of an image.

## References

[CR1] Dorigo M, Gambardella LM (1997). Ant colony system: a cooperative learning approach to the traveling salesman problem. IEEE Trans Evol Comput.

[CR2] Dorigo M, Stutzle T (2004). Ant colony optimization.

[CR3] Dorigo M, Maniezzo V, Colorni A (1991) Positive feedback as a search strategy, Technical Report. Politechnico di Milano, Italy, No. 91–016

[CR4] Li X, Orchard MT (2001). New edge-directed interpolation. IEEE Trans Image Process.

[CR5] Rukundo O, Cao HQ (2012). Nearest neighbor value interpolation. Int J Adv Comput Sci Appl.

[CR6] Rukundo O, Huang MH, Cao HQ (2011) Optimization of bilinear interpolation based on ant colony algorithm. In: Proceedings of the 2nd international conference electrical and electronics engineering, Macao, pp 571–580

[CR7] Tian J, Ma LH, Yu WY (2011). Ant colony optimization for wavelet-based image interpolation using a three-component exponential mixture model. J Expert Syst Appl.

